# Unraveling the Antimicrobial Effectiveness of *Coridothymus capitatus* Hydrolate against *Listeria monocytogenes* in Environmental Conditions Encountered in Foods: An In Vitro Study

**DOI:** 10.3390/microorganisms10050920

**Published:** 2022-04-27

**Authors:** Francesco Buccioni, Chiara Purgatorio, Francesca Maggio, Stefania Garzoli, Chiara Rossi, Luca Valbonetti, Antonello Paparella, Annalisa Serio

**Affiliations:** 1 Faculty of Bioscience and Technology for Food, Agriculture and Environment, University of Teramo, Via R. Balzarini 1, 64100 Teramo, Italy; fbuccioni@unite.it (F.B.); cpurgatorio@unite.it (C.P.); fmaggio@unite.it (F.M.); crossi@unite.it (C.R.); lvalbonetti@unite.it (L.V.); apaparella@unite.it (A.P.); 2 Department of Chemistry and Technologies of Drug, Sapienza University, Piazzale Aldo Moro 5, 00185 Rome, Italy; stefania.garzoli@uniroma1.it

**Keywords:** *L. monocytogenes*, *Coridothymus capitatus*, hydrolate, biopreservative, Phenotype Microarray, CLSM, sublethal concentrations

## Abstract

The increased resistance of bacteria to antimicrobials, as well as the growing interest in innovative and sustainable alternatives to traditional food additives, are driving research towards the use of natural food preservatives. Among these, hydrolates (HYs) have gained attention as “mild” alternatives to conventional antimicrobial compounds. In this study, the response of *L. monocytogenes* ATCC 7644 exposed to increasing concentrations of *Coridothymus capitatus* HY (CHY) for 1 h at 37 °C was evaluated by means of Phenotype Microarray, modelling the kinetic data obtained by inoculating control and treated cells into GEN III microplates, after CHY removal. The results revealed differences concerning the growth dynamics in environmental conditions commonly encountered in food processing environments (different carbon sources, pH 6.0, pH 5.0, 1–8% NaCl). More specifically, for treated cells, the lag phase was extended, the growth rate was slowed down and, in most cases, the maximum concentration was diminished, suggesting the persistence of stress even after CHY removal. Confocal Laser Scanner Microscopy evidenced a diffuse aggregation and suffering of the treated cells, as a response to the stress encountered. In conclusion, the treatment with HY caused a stressing effect that persisted after its removal. The results suggest the potential of CHY application to control *L. monocytogenes* in food environments.

## 1. Introduction

*Listeria monocytogenes* is a Gram-positive foodborne pathogen and causative agent of listeriosis. In 2020, more than 1870 cases of listeriosis were reported in the European Union (EU), with a stable trend in the years 2016–2020. The fatality rate of around 13.0% makes it one of the most severe foodborne diseases under surveillance in European countries [1]. Being ubiquitous, halotolerant and psychotropic, *L. monocytogenes* is widely distributed in the environment, and can contaminate a wide variety of matrices, such as foods and industrial surfaces. Among the main problems in the management of *L. monocytogenes* contamination is its removal from food-contact surfaces, due to its persistence, particularly in different food manufacturing environments. In fact, this microorganism can form biofilms on food-contact surfaces, acting as a potential source of cross-contamination of food products [2]. In the past decades, different antimicrobials have been employed to avoid or minimize *L. monocytogenes* growth. However, misuse and overuse of biocides have raised concerns over the emergence of microbial resistance [3]. In particular, the resistance developed by *L. monocytogenes* increases its ability to survive in hostile environments, as a consequence of adaptation to different stressors encountered along the food chain.

The increased microbial resistance to biocides, as well as the renewed interest of consumers in innovative and sustainable alternatives to synthetic food preservatives, have driven research towards the use of natural antimicrobial substances, the so-called “biopreservatives”. Biopreservatives are those antimicrobial compounds of plant, animal, and microbial origin that have been used as additives in food products without any adverse effects on human health [4]. Within the group of biopreservatives of plant origin, the most commonly used are Essential Oils (EOs), aromatic compounds extracted by distillation from different plant tissues. EOs, especially those rich in monoterpenes, demonstrate high antimicrobial efficacy and antioxidant features [5]. Among these, *Coridothymus capitatus* EO exerts strong antimicrobial effectiveness due to a high concentration of carvacrol [6,7]. Unfortunately, some difficulties in the use of EOs have been reported, mainly due to their low water solubility, high volatility, strong odor and flavor [8]. Hydrolates (also called hydrosols) can represent a “mild” and effective alternative to EOs. Hydrolates are basically composed by the condensing water in the EO distillation process, in which small amounts of volatile oil components, usually in concentrations lower than 1 g/L, are dispersed [7]. Their hydrophilic nature, together with their gentle sensory profile, makes HYs much more versatile than EOs for some applications [5,9]. Moreover, as a by-product of the EO production, they are cheaper and so applicable in a broader range of fields. Although in recent years the use of HYs as antimicrobials has been spreading, there is still a lack of information about their potential and safe use, particularly in food environments. Moreover, even though HY features represent a promising antimicrobial strategy in the food chain, it must be considered that a complex food can interact with the phenolic compounds and other active molecules contained in the natural antimicrobials, thus reducing the effect of the dose applied. As a consequence, only sublethal concentrations of biopreservative can come into contact with microbial cells, with unknown effects. Recently, we applied the Phenotype Microarray technology to investigate the effect of sublethal concentrations of EOs on *L. monocytogenes* cells, revealing interesting details on cell response [10]. 

In light of these considerations, the present study aimed to determine the effect of different concentrations of CHY on *L. monocytogenes* growth, to understand the effectiveness of HYs as biopreservatives in conditions encountered in food matrices.

In our work, the Omnilog system was used to study the growth potential of *L. monocytogenes* cells, previously exposed to CHY sublethal concentrations, in the presence of different environmental conditions and stressors commonly found in a variety of food products and environments, including carbon sources, organic acids, different salt concentrations and a range of acidic pH values. In fact, when applied in this kind of study, the Phenotype Microarray technology provides the opportunity to investigate the bacterial adaptive response in food-like conditions. 

## 2. Materials and Methods

### 2.1. Hydrolate

The experiments were conducted with a commercial, food-grade *Coridothymus capitatus* hydrolate, kindly provided by Exentiae S.r.l. (Catania, Italy). The CHY was stored at 4 °C in a dark bottle until the analysis. Therefore, CHY was diluted to 500, 400, 350, 300, 250 µL mL^−1^ with 10 mM Phosphate Buffer Saline (PBS) solution, pH 7.4.

### 2.2. Head Space GC-MS Analysis

To describe the chemical profile of the CHY vapor phase, about 2 mL of HY were placed in a 20 mL vial sealed with a headspace PTFE-coated silicone rubber septa and cap. For the analysis, a Headspace Turbomatrix 40 (PerkinElmer, Waltham, MA, USA) autosampler connected to a gas chromatograph with an FID (flame ionization) detector coupled directly to a mass spectrometer Clarus 500 model (Perkin Elmer Waltham, MA, USA) was used. The operative conditions, such as temperature and equilibration time, were optimized. The GC was equipped with a Varian Factor Four VF-1 capillary column. The carrier gas employed was helium at a flow rate of 1 mL/min. The Electron Impact-Mass Spectrometer (EI-MS) mass spectra were recorded at 70 eV (EI) and were scanned in the range 40–500 *m*/*z*. The ion source and connection parts temperature was 220 °C.

The oven temperature program was: from 60 °C ramped up to 220 °C at a rate of 6 °C min^−1^, and finally isothermal at 220 °C for 10 min. The identification of components was performed by matching their mass spectra with those stored in the Wiley and NIST 02 mass spectra libraries database. Moreover, for C_8_–C_30_ aliphatic hydrocarbons and the linear retention indices (LRIs) were calculated and compared with available retention data reported in the literature. Relative percentages of all identified components were obtained by peak area normalization from GC-FID chromatograms without the use of an internal standard or correction factors and were expressed in percentages. The analysis was performed three times.

### 2.3. Bacterial Strain

The study was conducted on type strain *L. monocytogenes* ATCC 7644, isolated from humans and belonging to serogroup 1/2c. It was first cultivated on Brain Heart Infusion (BHI) agar plates (Liofilchem, Roseto degli Abruzzi, Italy) overnight at 37 °C. One colony was inoculated for preculture in BHI broth (Liofilchem) at 37 °C for 18 h to obtain a fresh working culture at the beginning of the stationary phase. Bacteria were then harvested by centrifugation for 5 min at 13,000 rpm (Eppendorf centrifuge 5415D, Hauppauge, NY, USA) and washed twice with 10 mM PBS (pH 7.4) [11]. 

### 2.4. Exposure of L. monocytogenes Cells to Sublethal CHY Concentrations and Phenotype Microarray Determination

The effect of CHY on *L. monocytogenes* ATCC 7644 cells was determined through the OmniLog GEN III 96-wells microplates (Biolog Inc., Hayward, CA, USA) containing 71 carbon source utilization assays and 23 chemical sensitivity assays. A test tube was prepared in 16 mL 10 mM PBS (pH 7.4), containing about 5 × 10^8^ CFU mL^−1^ and CHY at the final concentration of 500, 400, 350, 300 and 250 µL mL^−1^. A control tube was prepared similarly, but without CHY treatment and with a corresponding volume of PBS. The tubes were then vortexed and incubated at 37 °C for 1 h. After incubation, cells were washed three times with 10 mM PBS (pH 7.4) to eliminate CHY residues and then collected in sterile tubes containing 16 mL IF-a fluid (Biolog, Hayward, CA, USA). The samples were subsequently adjusted to a final transmittance value of around 95%. Finally, one GEN III microplate for each tube was inoculated and incubated in the OmniLog incubator (Biolog, Hayward, CA, USA) at 37 °C for 72 h. Bacterial growth was monitored by the OmniLog reader by scanning the plates every 15 min. The analyses were repeated three times.

### 2.5. Data Analysis

In OmniLog Phenotype Microarray, the utilization of the substrates is assessed and measured as cell respiration, as determined by the grade of color development produced by the NADH reduction of a tetrazolium-based redox dye [12]. In this light, data from GEN III plates OmniLog readings were elaborated via Kinetic software (Biolog, Hayward, CA, USA) to obtain the parameters of microbial growth and images of the growth curves. The mean of the three biological repetitions was calculated and next, data were processed by fitting the growth curve by means of *Baranyi and Roberts* models [13] with DMFit software (available at www.combase.cc (accessed on 24 March 2022). 

The statistical data analysis was performed using XLSTAT statistical and data analysis solution (Addinsoft 2021). Analysis of variance with Tukey’s post-hoc test allowed to determine statistically significant differences among the effects of the different CHY concentrations on *L. monocytogenes* ATCC 7644 %CSH.

### 2.6. Confocal Laser Scanning Microscopy (CLSM)

Evaluation of the physiological response of *L. monocytogenes* ATCC 7644 to the different treatments with CHY concentrations was evaluated using CLSM analysis by means of a Nikon A1R confocal imaging system microscope, controlled by the Nikon NIS Elements software ver. 4.40 and equipped with a Plan Apo lambda 100 × oil objective (Nikon Corporation, Tokyo, Japan). In the treated samples, cells were exposed to 500, 450, 400, 350, 300, and 250 µL mL^−1^ of CHY, while a control sample was obtained without CHY treatment and after incubation for 1 h at 37 °C. After that, cells were washed three times with 10 mM PBS (pH 7.4) to eliminate CHY residues. The CLSM analysis was carried out using 5-Carboxyfluorescein Diacetate (5-cFDA) and Propidium iodide (PI) as fluorescent dyes (Molecular Probes, Eugene, OR, USA). Samples were subsequently treated with 50 μM cFDA and then incubated at 37 °C for 10 min in the dark to allow the intracellular enzymatic conversion from cFDA to cF. Then, cells were washed twice with 10 mM PBS (pH 7.4) to remove the cFDA excess. After that, PI 30 μM was added to the samples, then incubated for 15 min in ice in the dark to let the dye bind the DNA of the damaged cells. Next, cells were washed twice again with 10 mM PBS (pH 7.4) to remove PI excess and were resuspended. Ten microliters of the bacterial suspension were placed on glass chambers (Nunc; Thermo Fisher Scientific, Waltham, MA, USA). The cFDA was observed in FITC Channel with λ_exc_ = 485 nm and λ_em_ = 520 nm [14], and PI in TRITC Channel with λ_exc_ = 561.5 nm and λ_em_ = 595/50 nm [2].

### 2.7. Cell Surface Hydrophobicity (%CSH) Assay

The %CSH assay was performed according to the procedure described by *Rossi* et al. (2018) [15]. The bacterial strain was standardized at an OD_600nm_ = 1.0, which corresponds approximately to 10^9^ CFU mL^−1^. Therefore, different samples were prepared depending on the CHY concentrations (250, 300, 350, 400, and 500 µL mL^−1^) including a control sample without CHY treatment, and incubated for 1 h at 37 °C. After that, cells were washed twice and suspended in 10 mM PBS (pH 7.4) added with a 2 M ammonium sulfate solution (Sigma-Aldrich, Milan, Italy). Next, hexadecane (99%, Sigma-Aldrich, Milan, Italy) was added to the bacterial suspension in a 1:7 ratio, homogenized for 1 min and stored for 20 min at room temperature in order to obtain the two-phase separation. The plates were then submitted to absorbance measurement at OD_590nm_ by means of a MicroStation (Biolog, Hayward, CA, USA). The absorbance values were called *Ai* and *Af* for the samples before and after the addition of hexadecane 99% to suspension, respectively. The relative cell surface hydrophobicity (%CSH) of the strain was determined as reported in Equation (1) [16]:

[(*Ai* − *Af*) × 100]/*Ai* (1)

Results were reported for the different CHY concentration and expressed as percentage reduction of %CSH.

## 3. Results

### 3.1. Head Space GC-MS Analysis

**Figure 1:**
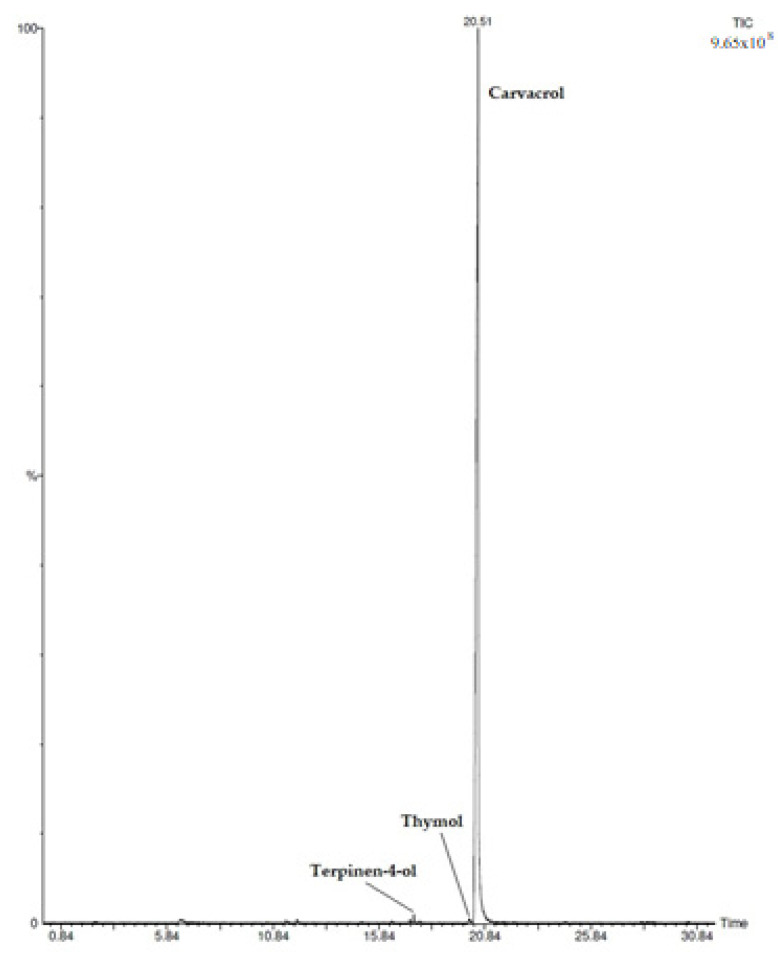
HS/GC-FID Chromatogram of *Coridothymus capitatus* Hydrolate.

In *C. capitatus* hydrolate, 6 compounds were identified among which carvacrol was the most abundant (98.9%) (Figure 1). Other minor compounds were identified and they ranged from 0.1% to 0.4%, as reported in Table 1.

### 3.2. Effect of C. capitatus HY Treatment on L. monocytogenes Growth in Presence of Different Substrates

After being exposed to different concentrations of CHY, *L. monocytogenes* ATCC 7644 cells were resuspended in the singular substrates contained in GEN III microplates (Biolog Inc., Hayward, CA, USA). After incubation at 37 °C for 72 h, the data were elaborated to obtain the growth dynamics and parameters of *L. monocytogenes* ATCC 7644 and to evaluate the microbial response in the different substrates. As shown in Figure S1, the treatment with CHY first had an effect on the lag phase, as a progressive extension was observed when cells were exposed to progressively increasing CHY quantities. In fact, for cells exposed to 250 µL mL^−1^, the growth profiles were almost superimposable to the control one, while differences were highlighted when CHY concentrations increased. The lag phase extension was clearly appreciable in cells exposed to 350 and 400 µL mL^−1^ in different substrates such as glucose (well C01), lactose (B02), trehalose (A04), cellobiose (A05), mannitol (C02) and fructose (C03). Except for the lag phase, the growth dynamics were similar for control and treated cells. On the contrary, after exposure to 500 µL mL^−1^ of CHY, the treated cells were unable to grow, and therefore this concentration was considered the Minimal Inhibitory Concentration (MIC). Focusing on the environmental conditions that can be encountered in food products, results regarding the growth parameters obtained at NaCl concentrations of 1, 4 and 8% are reported in Table 2, while those obtained at pH 6.0 and 5.0 are reported in Table 3. After the cells’ exposure to 500 µL mL^−1^ CHY, the software was unable to model the curves, and the growth parameters could not be calculated, indicating no growth. This concentration was therefore considered the MIC. As can be inferred from the tables, in the same conditions, the lag phase was progressively extended when the previous treatment was more intense, and the elongation was more noticeable as the environmental conditions became harder (pH 5.0 and 8% NaCl). In some cases, the final growth value (which is the maximum growth value reached by the cells) obtained by the modelling was slightly higher in treated than in control cells.

### 3.3. Effect of C. capitatus HY Treatment on L. monocytogenes Cells

The effect of exposure of *L. monocytogenes* ATCC 7644 cells to CHY for 1 h at 37 °C was observed through CLSM analysis. Cells were treated with double-staining assays. The first was cFDA, which is a cell permeant and can serve as a viability probe by measure of bacterial enzymatic activity. Through the hydrolysis of the diacetate group, cFDA is converted into fluorescent Carboxyfluorescein (CF) by intracellular non-specific esterase. The CF has a negative charge, which leads to retention within the cells and thus to the possibility to detect esterase-active bacterial cells [17]. The second probe was PI, a fluorescent intercalating that can bind DNA but is only able to pass the cytoplasmic membrane when it is damaged and not when it is intact [18]. Therefore, it can be exploited to observe damaged or dead cells. 

**Figure 2:**
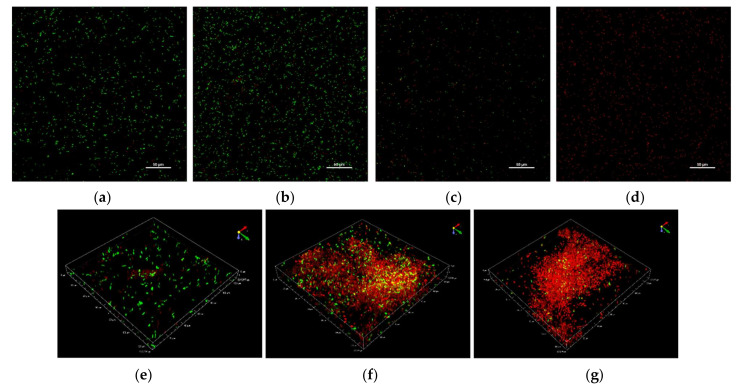
Representative CLSM images of *L. monocytogenes* ATCC 7644 cells exposed to CHY concentrations for 1 h at 37 °C. Control sample (**a**), 250 µL mL^−1^ (**b**), 350 µL mL^−1^ (**c**), and 500 µL ml^−1^ (**d**) of CHY. Cellular aggregation in presence of 250 µL mL^−1^ (**e**), 350 µL mL^−1^ (**f**), and 500 µL ml^−1^ (**g**) of CHY.

**Figure 3:**
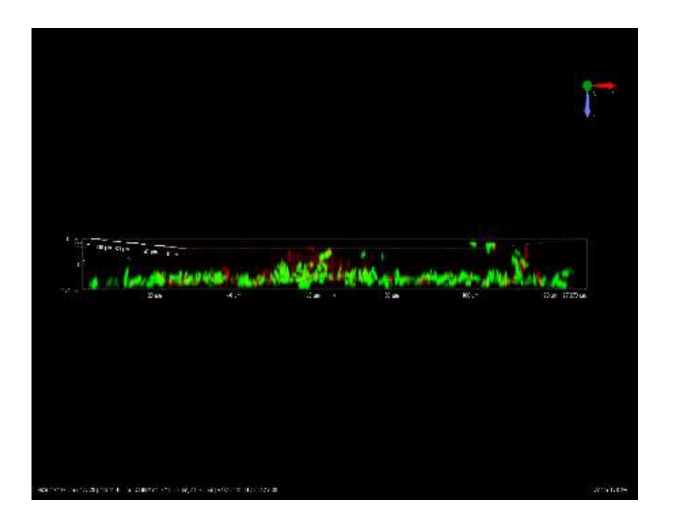
Representative CLSM image of *L. monocytogenes* ATCC 7644 cellular aggregates after exposure to 250 µL mL^−1^ CHY for 1 h at 37 °C.

Given the dose-dependent trend commonly observed after the treatment with CHY, in the following paragraphs, only the results of CHY concentrations of 250, 350 and 500 µL mL^−1^ have been shown. In Figure 2, cells not exposed (control samples, Figure 2a) and exposed to 250 (Figure 2b,e), 350 (Figure 2c,f) and 500 (Figure 2d,g) µL mL^−1^ of CHY were shown. These results evidenced that in the control sample, as well as in the presence of 250 µL mL^−1^ CHY treatment, most cells were alive (green), while only a few were damaged or dead (red). Although the untreated cells appeared as single cells, the exposition to CHY 250 µL mL^−1^ determined the presence of small cell aggregates of green color (alive cells) (Figure 3). However, in the presence of greater CHY concentrations, a dose-dependent increase of damaged or dead cells (red according to PI staining) was observed. Similarly, cell aggregation increased in line with the concentration of CHY used in the treatment. In fact, in the presence of the maximum concentration (500 µL mL^−1^), only aggregates of red cells were observed, suggesting an important stress condition and cell damage. 

**Figure 4:**
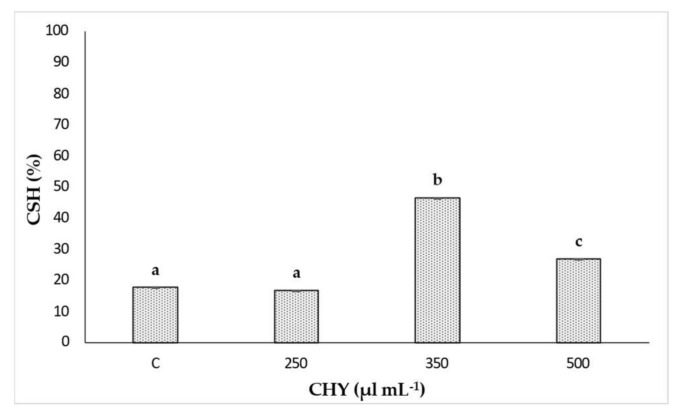
Cell surface hydrophobicity (%CSH) values observed for *L. monocytogenes* ATCC 7644 after exposure to 250, 350 and 500 µL mL^−^^1^ of CHY for 1 h at 37 °C. Control (C) corresponds to the untreated sample. Results are means of three biological repetitions and the bars indicates the standard deviations. Different letters indicate significant differences (*p* < 0.05) on %CSH among treatments with three CHY concentrations.

At the same time, the %CSH assay allowed the evaluation of the hydrophobicity of *L. monocytogenes* ATCC 7644 cells after CHY treatments. The results of %CSH after the exposition to 250, 350 and 500 µL mL^−1^ of CHY are depicted in Figure 4. The cell hydrophobicity was affected by the tested CHY concentration. In the presence of the minimum concentration (250 µL mL^−1^), no significant differences were observed compared to the control. On the contrary, a clear and significant (*p* < 0.05) cell hydrophobicity increase was detected in the presence of 350 µL mL^−1^ CHY. Finally, treatment with CHY 500 µL mL^−1^ significantly reduced (*p* < 0.05) the cellular hydrophobicity.

## 4. Discussion

The use of HYs as antimicrobials is gaining in importance in food applications because it has several advantages; nevertheless, some important aspects still need to be clarified. In particular, the reduced concentration of bioactive compounds compared to the relative EOs has led to conflicting opinions regarding their antimicrobial effectiveness. Moreover, to optimize the application in food systems, it is fundamental to comprehend how microbial cells behave when facing sublethal concentrations of antimicrobials. In this light, this study aimed to investigate the effect of the exposure to sublethal concentrations of *C. capitatus* hydrolate for 1 h at 37 °C. The cell response was evaluated in the presence of carbon sources and environmental conditions encountered in foods, by means of Omnilog GEN III microplates. First of all, the activity of CHY alone was observed (well A1) (Figure S1a–e), revealing that increasing concentrations determined an increasing extension of *L. monocytogenes* ATCC 7644 lag phase. This pattern cannot be explained by the cells’ natural death rate alone, because of the difference between treated and untreated samples, and the flattening of the growth curves in samples treated with lethal concentration of CHY (500 µL mL^−1^). The lag phase is commonly recognized as a preparative and adaptive phase for microorganisms; thus, its longer extension after the exposure of cells to CHY demonstrates that the hydrolate causes an unfriendly condition for *L. monocytogenes* growth. As a consequence of the stressing events determined in the cells by the increasing CHY quantities, the cells’ growth was progressively hampered [19,20]. Although the maximum growth rate was generally affected as well, it has to be underlined that, in some cases, the maximum growth value reached by the treated cells overtakes the untreated ones, notwithstanding the increase of the lag phase. This phenomenon is quite spread out among microbial species, and even more in *L. monocytogenes*: in fact, bacteria subjected to sublethal stresses may undergo physiological and genetic regulatory changes that, once recovered, may enhance their ability to survive hostile conditions [21]. In detail, a kind of stimulating effect on the growth, after a lag phase extension, has been already observed after treatment of cells with sublethal concentrations of essential oils [22] and could therefore be common to hydrolates as well. 

Then, the behavior of CHY treated cells in the presence of different pH values and NaCl concentrations, normally occurring in food, was analyzed. Still confirming the lag phase extension proportional to the increasing concentration of CHY, the presence of NaCl in the solution also exerted the same effect. In fact, combining the pre-exposure to 300 µL mL^−1^ CHY with 1% NaCl in the microplate well, the lag phase was equal to the one observed for cells growing in 8% NaCl alone (Table 2). Sodium chloride is often used in the food industry to control bacterial growth and extend shelf life [23]. *L. monocytogenes* is a halotolerant microorganism, meaning that it can tolerate NaCl percentages up to 10%, even though the growth rate decreases when the salt concentration increases [23]. The adaptation of *L. monocytogenes* to osmotic stress is mainly based on two different mechanisms, called primary and secondary responses: during the primary response, the cells enhance the entrance of potassium cations and glutamate through the membrane, followed by the uptake of osmoprotectants such as glycine betaine and carnitine, representing the secondary response [23,24]. The results obtained in this study suggested that even a pretreatment with low CHY concentrations produces stress on bacterial cells, affecting their ability to face the high NaCl percentage. 

Another food preservation strategy widely employed to extend the shelf life of food is the lowering of pH, commonly by means of organic acids. In detail, they act as antimicrobials, inhibit the polyphenol oxidase activity and prevent enzymatic browning in several food products [25]. The pH values (6.0 and 5.0) considered in this work are common in a variety of fresh foods, milk and dairy products, fish, meat, fruits and vegetables [23] and have been evaluated to simulate another possible scenario normally encountered in the food industry. These values normally sustain the growth of *L. monocytogenes,* which can tolerate moderate pH-related stresses, thanks to the presence of atypic branched-chain fatty acids. In detail, *L. monocytogenes* cells grown in the presence of various acids have been shown to incorporate more saturated fatty acids and less branched-chain fatty acids into their membrane, thus decreasing membrane fluidity in response to acidic stress [25,26]. Our results evidenced how an increasing CHY concentration produced a dose-dependent effect on the bacterial lag phase. As reported in Table 3, at pH 5.0, the lag phase extension was higher (8.24–35.45 h) than the corresponding concentration at pH 6.0 (1.09–14.28 h). This lag phase extension confirms the stress condition encountered by the bacterium, thus suggesting that the CHY pre-exposition also affects the bacterial tolerance to acidic stress.

The bioactive compounds contained in CHY are responsible for its antimicrobial effect. Carvacrol was the principal component (98.9%) revealed in the applied hydrolate (Table 1). This molecule is characterized by the presence of a hydroxyl group and a system of delocalized electrons [7,27]. These chemical structures allow carvacrol to act as a proton exchanger, which is able to reduce the transmembrane gradient. The consequence is the collapse of the proton motive force and the depletion of the ATP pool, which can lead to cell death [7,27]. In our case, exposure for 1 h at 37 °C at CHY concentrations between 250 and 400 µL mL^−1^ was not sufficient to inactivate the cells, but it probably disturbed the transmembrane gradient and hampered the energy production, thus leaving the cells with lower amounts of ATP, which required instead a response to stressing conditions. Moreover, although in very low amounts (about 0.4% of the total composition), terpinen-4-ol is also contained in CHY. This monoterpenic alcohol is the main component of tea tree oil and exerts antimicrobial and antibiofilm effects as a consequence of damage produced in the cytoplasmic membrane [28]. Furthermore, borneol and thymol, contained in CHY in even lower amounts, are commonly found in different essential oils and hydrolates with antimicrobial effects, such as *Lavandula angustifolia* and *Thymus vulgaris*, respectively [5]. It has to be considered that the chemotype of a hydrolate allows researchers to comprehend which kind of application could be suitable; nevertheless, the minor compounds are also essential to enriching and broadening the spectrum of biological activity [5].

Confocal Laser Scanning Microscopy, applied to evaluate the effect of the exposure to CHY on cells’ integrity and viability, once again evidenced a dose-response effect, thus confirming the results already discussed. The cells treated with the lowest CHY concentration (250 µL mL^−1^) did not significantly differ from the untreated ones, and only a few dead or damaged cells were observed, while most were still alive. After exposure to 350 µL mL^−1^ of CHY, the number of damaged cells increased and was almost equal to the alive ones. Above this concentration, damaged cells exceeded the alive ones, and at the highest concentration, 500 µL mL^−1^, almost no more viable cells were observed. The increase in red cells is probably related to the high quantity of carvacrol in CHY that penetrates the cell membranes, thereby causing their damage and progressive disruption [14]. An interesting aspect revealed by CLSM was the formation of aggregates: the reason behind the formation of these structures (the so-called ‘auto-aggregates’) can be identified in the intrinsic HY properties: in fact, despite being mostly composed of water, they still contain essential oil components that can exert a hydrophobic action, hence favoring cell adhesion and aggregation [5]. Some authors identified in bacterial aggregation a defensive mechanism against antibiotics and disinfectants. Antimicrobials trigger cell aggregation, as cells try to reduce the specific surface in contact with the antimicrobial solution to increase their survival possibilities [26]. Auto-aggregation of *L. monocytogenes* is a phenomenon that hardly occurs when cells are cultured alone or in an optimal state. On the contrary, under stressful conditions, such as nutritional starvation, microbial co-culture or challenges with an antimicrobial treatment, *L. monocytogenes* auto-aggregation occurs as a result of a complex reaction network that involves *quorum sensing*, flagellar assembly and changes in metabolic pathways (as the two-component system, the glycolysis/gluconeogenesis pathway, the ABC transporters, and the phosphotransferase system) [29]. The cell surface characteristics of microorganisms play an important role in various processes including aggregation, adhesion to surfaces, biofilm formation, uptake of chemicals or antimicrobial agents [15]. Even though cellular aggregation and cell hydrophobicity in *L. monocytogenes* are necessary for adhesion, the relationship between them is not yet completely clear. Changes in bacterial hydrophobicity may alter surface tensions among bacterial cells and result in a tendency of cells to attach to each other and form aggregates [30]. This could confirm the effect of CHY treatment on *L. monocytogenes* ATCC 7644. In fact, an increase in %CSH values was observed after exposure to an increasing of CHY concentration, up to 350 µL mL^−1^ (Figure 3). The presence of phospholipids was in part responsible for the high CSH, also contributing to the increased sensitivity to surfactants, hydrophobic antibiotics, and cationic compounds [31]. Thus, the higher the cellular hydrophobicity, the higher the sensitivity to the antimicrobial compounds. However, the %CSH results in the presence of the highest concentration of CHY (500 µL mL^−1^) (Figure 3), did not show an association with the higher aggregation observed with CLSM analysis (Figure 2). As a confirmation of our results, other authors [32] have reported that *L. monocytogenes* ATCC 7644, in presence of cranberry extract, did not show a linear relationship between the increase of cell surface hydrophobicity and auto-aggregation. The authors argued that the association between cell hydrophobicity and aggregation in the presence of environmental stresses may be a strain-dependent trait. 

## 5. Conclusions

In this study, Phenotype Microarray technology was applied to evaluate the response of *L. monocytogenes* ATCC 7644 cells to *Coridothymus capitatus* hydrolate. A single exposure to increasing concentrations of *C. capitatus* HY influenced the growth dynamics in the presence of different substrates commonly found in food products, with a dose-dependent response. Notwithstanding the ability of *L. monocytogenes* to tolerate moderate NaCl concentrations (up to 10%), the pre-exposure of the cells to CHY restored the antimicrobial effect of NaCl, starting from a 1% concentration. Furthermore, the pre-exposure of cells to CHY was sufficient to cause evident changes in membrane hydrophobicity and in cellular spatial distribution, resulting in cellular auto-aggregation, which is considered a stress response mechanism, where the external cells are more exposed to antimicrobials, while preserving the internal ones.

In conclusion, this study confirms the effectiveness of CHY against *L. monocytogenes* ATCC 7644 and the possibility of inducing a persisting stress response in cells after its removal. The data suggest a promising use of CHY to control the contamination of *L. monocytogenes* in food processing environments. Moreover, the features of HYs, including the gentle sensory profile, the hydrophilic nature, and the lower cost compared to essential oils suggest their potential application in food formulations or in food processing environments (e.g., for sanitization of food-contact surfaces). Finally, in the “multi-drug resistance” era, the presented data underline the importance of studying how natural bioactive compounds affect the microbial response of pathogenic bacteria in order to hinder their resistance. 

## Figures and Tables

**Table 1:** Chemical composition (percentage mean value ± standard deviation) of CHY. The table displays: the components reported according to their elution order on apolar column, Molecular weight (MW); Molecular Formula (MF), the Linear Retention Indices measured on apolar column (LRI_m_); the Linear Retention Indices from literature (LRI_t_); mass-to-charge ratio (*m*/*z*); percentage mean values of *Coridothymus capitatus* vapor phase components (*C. c.*) (peak area %).

N°	Component	MW	MF	LRI_m_	LRI_t_	*m*/*z*	*C. c.* (Peak Area %)
1	*β*-pinene	136.2340	C_10_H_16_	972	969	93, 41, 69, 136	0.1 ± 0.02
2	borneol	154.2493	C_10_H_18_O	1151	1154	95, 110, 154	0.2 ± 0.02
3	terpinen-4-ol	154.2493	C_10_H_18_O	1178	1182	71, 111, 93, 154	0.4 ± 0.01
4	*α*-terpineol	154.2493	C_10_H_18_O	1180	1183	59, 93, 121, 136, 154	0.1 ± 0.00
5	thymol	150.2176	C_10_H_14_O	1290	1287	135, 150, 91	0.1 ± 0.00
6	carvacrol	150.2176	C_10_H_14_O	1310	1304	135, 150, 91	98.9 ± 0.04
	Sum						99.8

**Table 2:** Growth parameters of *L. monocytogenes* ATCC 7644 cells, previously exposed to increasing CHY concentrations, in presence of 1 (well B10), 4 (B11) and 8 (B12) % NaCl concentration. Results are obtained from the Baranyi and Roberts model. The table displays: Lag phase (h), Maximum growth rate (Omnilog unit/h) the final (Vf) cell density value (Omnilog unit), the R-squared (R^2^), the standard error (SE) and the assessment of the fitting model (Model).

NaCl Concentration	CHY (µL mL^−1^)	Lag Phase (h)	Maximum Rate (Omnilog Unit/h)	Vf (Omnilog Unit)	R^2^	SE	Model
1%	0 (Ctrl)	1.08 ± 0.16	50.22 ± 2.91	170.19 ± 1.56	0.996	4.24	Complete
1%	250	-	25.43 ± 1.28	149.94 ± 1.93	0.990	50.4	No lag
1%	300	5.37 ± 0.28	38.94 ± 3.68	169.75 ± 2.69	0.990	8.04	Complete
1%	350	13.15 ± 0.18	58.37 ± 5.52	191.33 ± 2.43	0.993	7.53	Complete
1%	400	14.88 ±0.13	39.87 ± 2.19	160.42 ± 0.66	0.996	4.15	Complete
1%	500	-	-	-	-	-	Unmodelable
4%	0 (Ctrl)	1.60 ± 0.12	34.64 ± 1.04	168.28 ± 1.07	0.999	2.46	Complete
4%	250	-	17.55 ± 0.68	143.01 ± 2.04	0.993	4.17	No lag
4%	300	8.18 ± 0.23	34.14 ± 2.86	159.09 ± 3.39	0.991	60.87	Complete
4%	350	17.63 ± 0.13	44.20 ± 2.45	185.64 ± 1.58	0.997	4.56	Complete
4%	400	19.30 ± 0.22	34.20 ± 3.10	151.04 ± 2.09	0.991	60.74	Complete
4%	500	-	-	-	-	-	Unmodelable
8%	0 (Ctrl)	2.03 ± 0.20	19.03 ± 0.59	161.92 ± 1.79	0.998	2.62	Complete
8%	250	-	11.26 ± 0.39	151.58 ± 1.98	0.991	4.90	No lag
8%	300	11.77 ± 0.77	11.62 ± 1.07	-	0.940	11.11	No asymptote
8%	350	30.72 ± 0.47	16.25 ± 1.73	148.68 ± 1.77	0.980	8.17	Complete
8%	400	33.46 ±0.20	16.50 ± 0.80	134.00 ± 0.92	0.995	3.55	Complete
8%	500	-	-	-	-	-	Unmodelable

**Table 3:** Growth parameters of *L. monocytogenes* ATCC 7644 cells, previously exposed to increasing CHY concentrations, in presence of pH 6.0 (well A11) and pH 5.0 (A12). Results are obtained from the Baranyi and Roberts model. The table displays: Lag phase (h), Maximum growth rate (Omnilog unit/h) the final (Vf) cell density value (Omnilog unit), the R-squared (R^2^), the standard error (SE) and the assessment of the fitting model (Model).

pH Value	CHY (µL mL^−1^)	Lag Phase (h)	Maximum Rate (Omnilog Unit/h)	Vf (Omnilog Unit)	R^2^	SE	Model
6.0	0 (Ctrl)	2.53 ± 0.08	105.08 ± 7.50	186.55 ± 1.46	0.997	4.60	Complete
6.0	250	1.09 ± 0.50	28.41 ± 2.88	168.79 ± 2.92	0.980	8.57	Complete
6.0	300	6.54 ± 0.23	62.51 ± 8.14	185.90 ± 4.84	0.987	9.31	Complete
6.0	350	13.36 ± 0.14	56.29 ± 3.79	220.15 ± 3.10	0.995	6.12	Complete
6.0	400	14.28 ± 0.19	46.67 ± 4.13	176.93 ± 3.31	0.991	6.60	Complete
6.0	500	-	-	-	-	-	Unmodelable
5.0	0 (Ctrl)	4.78 ± 0.21	56.60 ± 6.56	160.06 ± 2.25	0.990	7.23	Complete
5.0	250	8.24 ± 0.29	44.58 ± 6.58	162.51 ± 3.32	0.983	9.08	Complete
5.0	300	17.16 ± 0.24	45.93 ± 5.84	161.80 ± 4.19	0.982	7.97	Complete
5.0	350	22.69 ± 0.18	81.56 ± 13.86	189.28 ± 4.12	0.981	8.76	Complete
5.0	400	35.45 ± 1.18	4.98 ± 0.48	-	0.876	9.71	No asymptote
5.0	500	-	-	-	-	-	Unmodelable

## Data Availability

Data will be available on request due to restrictions (part of a project).

## Supplementary Materials

The following supporting information can be downloaded at https://www.mdpi.com/article/10.3390/microorganisms10050920/s1, Figure S1: Superimposition of the growth curves of *Listeria monocytogenes* ATCC 7644 not exposed and exposed to 250, 300, 350, 400 and 500 µL mL^−1^ of *C. capitatus* hydrolate.
