# Supplementary material for: Unraveling the Antimicrobial Effectiveness of Coridothymus capitatus Hydrolate against Listeria monocytogenes in Environmental Conditions Encountered in Foods: An In Vitro Study
Source: Microorganisms. 2022 Apr 27;10(5):920. doi: 10.3390/microorganisms10050920 (PMC9146057; doi:10.3390/microorganisms10050920)
Supplement: Supplementary file 1 [file microorganisms-10-00920-s001.zip › microorganisms-1692253-supplementary.pdf]

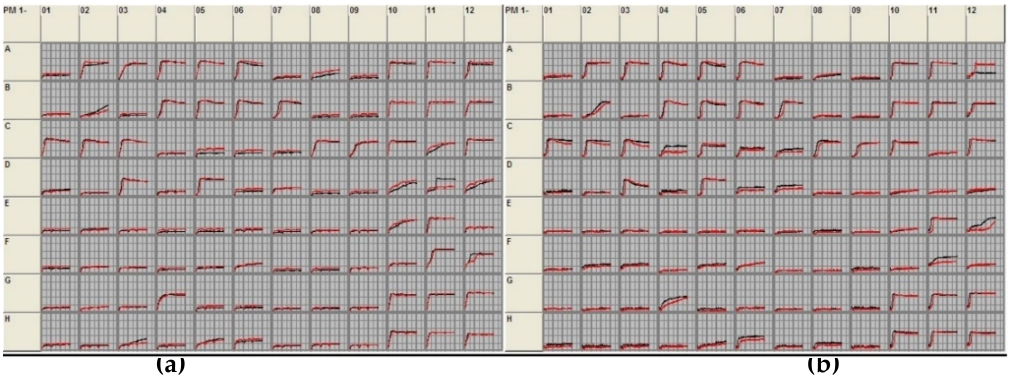

(a) (b)

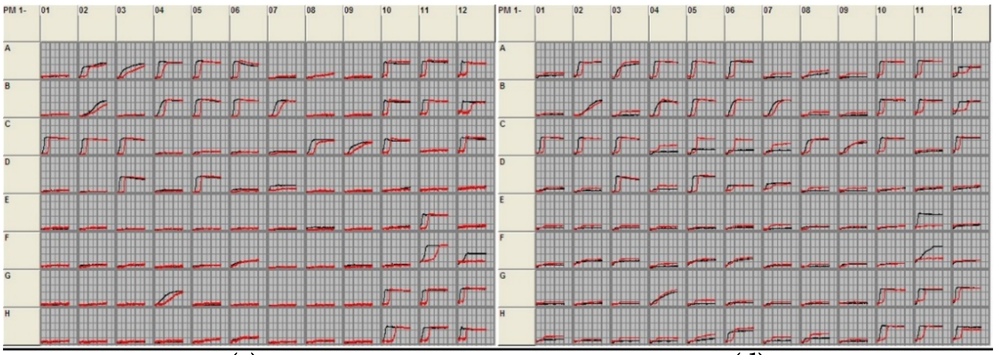

(c) (d)

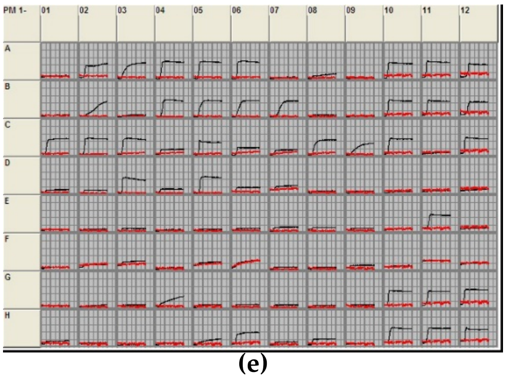

(e)

Figure S1. Superimposition of the growth curves (mean of three repetitions) of *L. monocytogenes* ATCC 7644 not exposed (control) and exposed to 250 (**a**), 300
(**b**) 350 (**c**), 400 (**d**), 500 (**e**) $\mu L\ mL^{-1}$ CHY (treated sample) for 1 h at 37°C. Each square represents a single microplate well. Black = growth curve of untreated cells; red= growth curve of treated cells. Legend: A1 no substrates, only CHY, A2 dextrin, A3 D-maltose, A4 D-trehalose, A5 Dcellobiose, A6 gentiobiose, A7 sucrose, A8 D-turanose, A9 stachyose, A10 positive control, A11 pH6, A12 pH5, B1 D-raffinose, B2 α-D-lactose, B3 D-melibiose, B4 β-methyl-D-glucoside, B5 D-salicin, B6 N-acetyl-D-glucosamine, B7 N-acetyl-β-D-mannosamine, B8 N-acetyl-β-D-galactosamine, B9 N-acetyl neuraminic acid, B10 1% NaCl, B11 4% NaCl, 3-methyl-glucose, C6 D-fucose, C7 L-fucose, C8 Lrhamnose, C9 inosine, C10 1% sodium lactate, C11 fusidic acid, C12 D-serine, D1 D-sorbitol, D2 D-mannitol, D3 D-arabitol, D4 myo-inositol, D5 glycerol, D6 D-glucose-6-PO4, D7 D-fructose-6-PO4, D8 D-aspartic acid, D9 D-serine, D10 troleandomycin, D11 rifamycin SV, D12 minocycline, E1 gelatin, E2 glycyl-Lproline, E3 L-alanine, E4 L-arginine, E5 L-aspartic acid, E6 L-glutamic acid, E7 Lhistidine, E8 L-pyroglutamic acid, E9 L-serine, E10 lyncomycin, E11 guanidine HCl, E12 Niaproof 4, F1 pectin, F2 D-galacturonic acid, F3 L-galactonic acid lactone, F4 D-gluconic acid, F5 D-glucuronic acid, F6 glucuronamide, F7 mucic acid, F8 quinic acid, F9 D-saccharic acid, F10 vancomycin, F11 tetrazolium violet, F12 tetrazolium blue, G1 p-hydroxy-phenylacetic acid, G2 methyl pyruvate, G3 D-lactic acid methyl ester, G4 L-lactic acid, G5 citric acid, G6 α-ketoglutaric acid, G7 D-malic acid, G8 L-malic acid, G9 bromo-succinic acid, G10 nalidixic acid, G11 lithium chloride,

G12 potassium tellurite, H1 tween 40, H2 γ-amino-butyric acid, H3 α-hydroxy-butyric acid, H4 β-hydroxy-butyric acid, H5 α-keto-butyric acid, H6 acetoacetic acid, H7 propionic acid, H9 formic acid, H10 aztreonam, H11 sodium butyrate, H12 sodium bromate.
